# Comparison of Mini-Percutaneous Nephrolithotomy and Flexible Ureteroscopy for Treating 1–2 cm Single Stones in Solitary Kidney: Outcomes and Renal Function Impact

**DOI:** 10.3390/jcm15052089

**Published:** 2026-03-09

**Authors:** Yuehan Yang, Zhongwei Jiang, Xike Mao, Lvwen Zhang, Zongyao Hao

**Affiliations:** 1 Department of Urology, The First Affiliated Hospital of Anhui Medical University, Hefei 230000, China; 2 Institute of Urology, Anhui Medical University, Hefei 230032, China; 3 Anhui Province Key Laboratory of Urological and Andrological Diseases Research and Medical Transformation, Anhui Medical University, Hefei 230032, China; 4 Department of Urology, The Third Affiliated Hospital of Shenyang Medical College (Shenyang 242 Hospital), Shenyang 110167, China; 5 Department of Urology, Shengjing Hospital of China Medical University, Shenyang 110000, China

**Keywords:** solitary kidney, renal stones, flexible ureteroscopy, mini-percutaneous nephrolithotomy, renal function, eGFR

## Abstract

**Objective**: The optimal surgical approach for 1.0–2.0 cm renal stones in solitary kidney patients remains controversial. This retrospective study compared mini-percutaneous nephrolithotomy (mPCNL) and flexible ureteroscopy (f-URS) outcomes in this vulnerable population. **Methods**: Between June 2018 and April 2024, 50 patients with solitary kidneys and 1.0–2.0 cm renal stones underwent either mPCNL (*n* = 26) or f-URS (*n* = 24). Outcomes included 3-month stone-free rate (SFR), complications (Clavien–Dindo classification), and renal function (serum creatinine, eGFR by CKD-EPI equation) at the baseline, 72 h, and 1 month. **Results**: Stone-free rates were comparable (mPCNL 96.2% vs. f-URS 91.7%, *p* = 0.157). The f-URS group demonstrated significantly less hemoglobin decline (2.2 ± 0.9 vs. 5.7 ± 2.4 g/dL, *p* < 0.001) and shorter hospitalization (4.1 ± 1.1 vs. 7.8 ± 1.6 days, *p* < 0.001). All Grade II complications (8.3%, requiring transfusion) occurred in the mPCNL group. At 1 month, serum creatinine decreased more with f-URS (15.4 ± 7.96 vs. 8.7 ± 4.23 μmol/L, *p* < 0.001), with greater eGFR improvement (16.7 ± 4.7 vs. 15.4 ± 5.2 mL/min/1.73 m^2^, *p* = 0.023). **Conclusions**: In this retrospective cohort, f-URS achieved comparable stone clearance to mPCNL alongside a superior early safety profile and better short-term renal functional preservation. These preliminary findings suggest that f-URS represents a viable nephron-sparing option for this high-risk population. However, these results are considered hypothesis-generating, and further prospective, long-term studies are required to evaluate the durability of these functional benefits.

## 1. Introduction

The management of urinary stones in solitary kidney patients is a complex clinical issue because of the potential complications associated with the increased risk of surgical treatment [1,2]. With increasing incidences of functional solitary kidneys—resulting from congenital anomalies or contralateral nephrectomy—the number of such patients has risen [3,4]. Therefore, treatment strategies must prioritize renal preservation to prevent irreversible functional decline [5,6].

Current urological guidelines and studies have reported that both mini-percutaneous nephrolithotomy (mPCNL) and flexible ureteroscopy (f-URS) are viable first-line interventions for patients with solitary renal stones [7,8,9,10,11]. Shi et al. evaluated 117 patients with solitary kidney who had undergone PCNL or RIRS for renal stones larger than 2 cm and concluded that PCNL offered fewer repeat procedures and comparable overall costs, supporting its use as a preferred option [12]. Lai et al. proved that RIRS is a safe and effective procedure for the treatment of renal stones in patients with solitary kidneys, with no adverse effects on renal function during follow-up [13]. Regardless of the chosen modality, maintaining or improving renal function while achieving high SFR remains a critical goal.

Prior research has primarily focused on larger stones (>2 cm), aiming to optimize procedural efficiency and minimize complications [11,12]. However, there is limited referential data on the safety and treatment outcomes of solitary renal stones smaller than 2 cm. This retrospective study aims to fill this gap by evaluating the treatment outcomes of mPCNL and f-URS for 1.0–2.0 cm solitary renal stones and renal function changes.

## 2. Materials and Methods

This retrospective study was conducted between June 2018 and April 2024 at a tertiary hospital. Initially, 86 consecutive patients with solitary kidney stones who underwent lithotripsy were recruited. After the screening, 50 patients were included in the final analysis; 26 received mPCNL, and 24 received f-URS. Written informed consent was obtained from all individual participants prior to their enrollment. The consent process involved detailed explanations of the study’s purpose, procedures, potential risks, and benefits, supported by standardized information sheets. All patient data were de-identified, coded, and stored in a secure, password-protected database accessible only to the principal investigators, in strict compliance with the Declaration of Helsinki and relevant data protection regulations.

### 2.1. Inclusion and Exclusion Criteria

**Figure 1:**
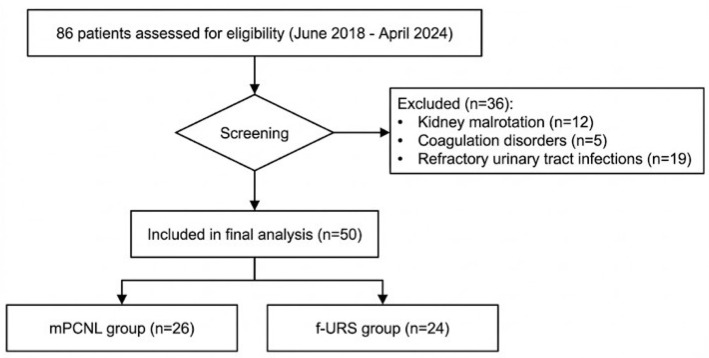
Patient flowchart detailing the study selection process.

A “solitary kidney” was defined as either congenital absence of one kidney, acquired loss due to nephrectomy, or presence of a non-functional contralateral kidney. The inclusion criteria: aged 18–80 years; presence of a single renal stone measuring 1.0–2.0 cm confirmed by non-contrast computed tomography. Exclusion criteria: presence of medical conditions constituting absolute contraindications to mPCNL or f-URS; inability to comprehend or comply with study requirements (Figure 1). Of the initial cohort, 36 patients were excluded based on these criteria, specifically due to kidney malrotation (*n* = 12), coagulation disorders (*n* = 5), and refractory urinary tract infections (*n* = 19).

### 2.2. Selection of Surgical Method

The surgical approach (mPCNL or f-URS) was determined based on the following factors: surgeon preference, patient preference, anatomical considerations, stone characteristics, institutional availability, and shared decision-making. Allocation was consecutive and non-randomized, with no predefined clinical algorithm or eligibility pathway for selecting between mPCNL and f-URS. Prior to surgery, the risks and benefits of both procedures were explained to the patients using standardized information sheets (Supplementary Table S1). All patients were fully informed and participated in the final decision-making process (i.e., a shared decision-making model).

### 2.3. Surgical Procedures 

f-URS: The procedure was performed under general anesthesia with the patient in the lithotomy position. Following successful anesthesia, a flexible digital ureteroscope (Innovex Medical Co., Ltd., Shanghai, China) (working channel 3.6 Fr) was advanced into the ureter. Ureteral access sheath (UAS) placement was attempted in all cases following guidewire insertion, with the decision based on preoperative imaging assessment of ureteral diameter and stone location; a 13 Fr UAS was routinely used to avoid elevated intrarenal pressure, facilitate repeated scope passage, and enable the passage of stone fragments. When UAS could not be advanced due to ureteral stricture or tortuous anatomy, the following stepwise approach was employed: (1) placement of a double-J stent (6 Fr or 7 Fr) for passive dilation with a planned second-stage procedure 2–4 weeks later; (2) downsizing to a smaller caliber sheath; or (3) proceeding with sheathless ureteroscopy when deemed safe. Intracorporeal lithotripsy was performed using a holmium:YAG laser (Shanghai Raykeen Laser Technology Co., Ltd., Shanghai, China) (20–30 W) with 200–272 μm laser fiber. For stones < 15 mm, a dusting technique was primarily employed (0.5–0.8 J, 10–15 Hz, long pulse); for stones ≥15 mm, a fragmentation approach was used (1.0 J, 20 Hz, 20 W) to create fragments suitable for basket extraction, with all visible fragments > 3 mm actively retrieved using a nitinol basket (1.9–2.2 Fr) (InnovEX Medical Co. Ltd., Shanghai, China). Continuous low-pressure irrigation was maintained using gravity-fed normal saline from a height of 60–80 cm, ensuring that intrarenal pressure remained <30 cmH_2_O, with irrigation temporarily ceased if excessive pelvic distension was visualized. A 6 Fr double-J stent was routinely placed postoperatively in all patients with solitary kidneys based on the following indications: ureteral injury, residual stone fragments, prolonged operative time (>90 min), or significant ureteral edema; stents were routinely removed 2–4 weeks postoperatively under local anesthesia. All patients underwent kidney–ureter–bladder (KUB) radiography on postoperative day 1, with non-contrast CT performed at 4–6 weeks post-stent removal to assess stone-free status (defined as no residual fragments or fragments < 2 mm).

mPCNL: Under general anesthesia, a 5 Fr ureteral catheter was inserted into the target ureter via ureteroscopy, and bladder drainage was established using a 16 Fr Foley catheter (Create Medic, Yokohama, Japan). The patient was then repositioned into the prone position. Percutaneous access was obtained under combined ultrasound and fluoroscopic guidance using an 18-gauge coaxial needle (Dermax Technology Limited, Shijiazhuang, Hebei, China) to puncture the selected calyx, with target calyx selection based on stone location and configuration, prioritizing lower pole access when feasible to minimize vascular injury risk, while upper or middle calyx puncture was performed when stone distribution required it. Tract dilation was performed with sequential fascial dilators up to 18 Fr. Single-tract access was achieved in the majority of cases, with multiple tracts (2 tracts) required in cases with complex stone burden or unfavorable anatomy. Stone fragmentation was carried out using holmium laser lithotripsy (20–40 W, 0.8–1.5 J, 15–30 Hz) as the primary lithotripsy modality, with pneumatic lithotripsy used as an adjunct when needed. The decision for nephrostomy tube placement versus tubeless approach was made intraoperatively based on the following criteria: tubeless mPCNL was performed when complete stone clearance, minimal bleeding, single-tract access, no collecting system perforation, and no planned second-look procedure were achieved; otherwise, a 14–16 Fr nephrostomy tube (Reborn medical, Zhuzhou City, Hunan, China) was placed and typically removed on postoperative days 2–3 if output was minimal and imaging confirmed no obstruction. At the conclusion of the procedure, a 6 Fr double-J ureteral stent was left in place. Transfusion was indicated when hemoglobin dropped >3 g/dL with hemodynamic instability or when hemoglobin fell below 7 g/dL, with selective angioembolization considered for persistent bleeding despite conservative management.

### 2.4. Standardized Perioperative Care

All patients received intravenous antibiotic prophylaxis (ceftriaxone 2 g or alternative based on culture sensitivities and allergies) 30–60 min prior to incision, with antibiotic therapy continued for 24–48 h postoperatively and extended based on intraoperative stone culture results or clinical signs of infection. Preoperative urine culture was mandatory for all patients within 1 week of surgery; active urinary tract infections were treated with culture-directed antibiotics for at least 7 days prior to surgery with repeat culture confirming sterility, and intraoperative stone fragments were sent for culture in all cases. Patients were discharged when meeting all of the following criteria: stable vital signs for ≥24 h, adequate pain control with oral analgesics (Visual Analog Scale < 4), absence of fever (temperature < 38 °C) for ≥24 h, satisfactory postoperative imaging (KUB showing acceptable fragment clearance and no significant hydronephrosis), and tolerating oral intake without nausea or vomiting.

### 2.5. Measurement of Characteristics

The comparative analysis between the mini-PCNL and f-URS groups encompassed the following domains: patient demographics, stone characteristics (laterality, maximum diameter, and density), perioperative outcomes (operative time, hospital stay, and stone-free rate), postoperative complications (graded by the Clavien–Dindo classification), and serial renal function evaluation.

#### 2.5.1. Primary Efficacy Outcome: Stone-Free Rate (SFR)

The stone-free rate served as the primary efficacy endpoint. Stone-free status was stringently defined as the absence of any detectable residual fragment (i.e., fragment size of 0 mm) on a low-dose, non-contrast computed tomography (CT) scan reconstructed at a 2-mm slice thickness. This confirmatory imaging was uniformly scheduled and performed at the 3-month postoperative follow-up for all enrolled patients.

#### 2.5.2. Perioperative and Safety Outcomes

Operative hemoglobin loss: Quantified as the mean absolute reduction in hemoglobin concentration (g/dL) measured at 24 h post-procedure relative to the preoperative baseline value. All adverse events occurring within a 30-day postoperative period were prospectively recorded and severity-graded according to the Clavien–Dindo classification system.

#### 2.5.3. Renal Function Evaluation

Given the critical importance of renal preservation in this solitary kidney cohort, function was assessed using a dual-marker approach: serum creatinine (Scr) and the estimated glomerular filtration rate (eGFR), the latter calculated using the Chronic Kidney Disease Epidemiology Collaboration (CKD-EPI) equation. In 2021, an updated CKD-EPI equation (CKD-EPI2021) that omits race as a variable was published and promptly recommended for clinical implementation by nephrology societies [14,15]. This revised formula estimates glomerular filtration rate (eGFR) based solely on serum creatinine level, age, and sex. Patients were categorized according to CKD stage (stages 1–5) defined by the National Kidney Foundation. The study cohort consisted of patients with an eGFR of ≥60 mL/min per 1.73 m^2^ (CKD stage ≤ 3).

To ensure transparency and clinical interpretability, absolute values for Scr (μmol/L) and eGFR (mL/min/1.73 m^2^) are reported for three prespecified timepoints: preoperative (baseline), 72 h postoperatively, and 1 month postoperatively. The change in renal function is expressed as the absolute difference between the postoperative and preoperative values. A negative ΔScr denotes a functional improvement (decrease in creatinine), whereas a positive ΔeGFR denotes a functional improvement (increase in filtration rate).

### 2.6. Statistical Analysis

All statistical analyses were performed using SPSS software (version 22.0; IBM Corp.). The present study included a total of 50 patients, a sample size determined by pragmatic considerations based on the available cohort of solitary kidney patients meeting the inclusion criteria during the study period. Given this sample size and the evaluation of multiple secondary outcomes, the study is acknowledged to have limited statistical power for detecting anything but large effect sizes, particularly for less frequent endpoints such as major complications. Continuous variables were assessed for normality using the Shapiro–Wilk test. Data with a normal distribution are presented as the mean ± standard deviation (SD) and were compared between the mini-PCNL and f-URS groups using the independent samples Student’s t-test. Non-normally distributed continuous variables were summarized as the median (interquartile range [IQR]) and compared using the Mann–Whitney U test. Categorical variables were reported as number (percentage) and compared using the Chi-square test or Fisher’s exact test, as appropriate.

To address potential confounding arising from the non-randomized, consecutive allocation of patients, we employed a dual strategy. First, baseline demographic and clinical characteristics were compared between groups to identify significant imbalances. Second, for key continuous outcomes (e.g., operative time, change in renal function), an analysis of covariance (ANCOVA) was performed where feasible, adjusting for identified baseline imbalances (e.g., stone size, density) to estimate adjusted between-group differences. A two-sided *p* value < 0.05 was considered statistically significant.

## 3. Results

### 3.1. Demographic and Clinical Data of the Study Population

A total of 50 patients with solitary kidney stones were included in the final analysis. Baseline demographic and clinical characteristics (including age, gender, BMI, comorbidities, and kidney characteristics), and stone characteristics (including side, size and density) are summarized in Table 1. No statistically significant differences were observed between the mPCNL and f-URS groups (all *p* > 0.05).

### 3.2. Perioperative Treatment Results and Safety Profile

As shown in Table 2, no significant difference was found in operative time between the two groups (*p* = 0.106). However, the mPCNL group exhibited a significantly greater decline in postoperative hemoglobin levels compared to the f-URS group (5.7 ± 2.4 g/dL vs. 2.2 ± 0.9 g/dL, *p* < 0.001). The length of hospitalization was significantly shorter in the f-URS group (4.1 ± 1.1 days vs. 7.8 ± 1.6 days, *p* < 0.001).

The stone-free rate (SFR) at the 3-month follow-up was comparable between the cohorts. The mPCNL group achieved an SFR of 96.2% (95% CI: 81.1–99.3%), while the f-URS group reached 91.7% (95% CI: 74.2–97.7%) (*p* = 0.157). Postoperative complications, graded by the Clavien–Dindo classification system, showed no significant difference in the overall rate between the groups (23.1% vs. 20.8%, *p* = 0.656), with 95% CIs of 11.0–42.1% and 9.2–40.5%, respectively.

In the mPCNL group, five complications were recorded: three cases of renal colic (Grade I), one case of self-limiting fever (body temperature > 38.0 °C, Grade I), and two cases of bleeding requiring blood transfusion (Grade II). No ureteral injuries occurred in this cohort. In the f-URS group, all five complications were Grade I self-limiting fevers, none of which required antibiotic treatment or led to secondary infection. Despite the routine use of a ureteral access sheath, no ureteral injuries or strictures were observed in the f-URS group during the follow-up period. No complications of Grade III or higher, septic shock, or mortality occurred in either cohort.

Given the pragmatic sample size of 50 patients, the study is acknowledged to be underpowered for detecting rare but clinically relevant adverse events such as urosepsis, acute kidney injury, or late stricture formation. These results are considered hypothesis-generating, suggesting a potential safety profile for f-URS that warrants validation in larger, prospective comparative trials.

### 3.3. Renal Function Changes

The primary metric for renal function evaluation was eGFR. Both groups showed a significant post-procedural increase in eGFR. The f-URS group demonstrated a significantly higher absolute improvement in eGFR (ΔeGFR) compared to the mPCNL group at 72 h (7.6 ± 3.73 vs. 6.1 ± 5.32 mL/min/1.73 m^2^, *p* = 0.044) and at 1 month (16.7 ± 4.7 vs. 15.4 ± 5.2 mL/min/1.73 m^2^, *p* = 0.023) (Table 3).

Correspondingly, serum creatinine (Scr) levels decreased in both cohorts. While baseline Scr levels were comparable (*p* = 0.543), the f-URS group achieved a significantly greater reduction in Scr (ΔScr) at 72 h (6.1 ± 4.64 vs. 3.6 ± 2.45 μmol/L, *p* = 0.024) and 1 month (15.4 ± 7.96 vs. 8.7 ± 4.23 μmol/L, *p* < 0.001).

To address potential selection bias arising from the non-randomized treatment allocation, an analysis of covariance (ANCOVA) was performed to estimate adjusted between-group differences. After adjusting for baseline stone diameter, stone density, and preoperative renal function (baseline eGFR or Scr), the f-URS group continued to demonstrate a statistically significant advantage in early functional recovery (Table 4).

At the 1-month follow-up, the adjusted mean difference in ΔeGFR was 1.28 mL/min/1.73 m^2^ (95%CI: 0.19–2.41; *p* = 0.023) in favor of the f-URS group. Similarly, the adjusted ΔScr at 1 month showed a significantly greater reduction in the f-URS cohort compared to the mPCNL cohort (adjusted mean difference: −6.75 μmol/L; 95%CI: −10.31 to −3.09; *p* < 0.001). While these differences were statistically significant, the absolute magnitude of the eGFR improvement remained modest, suggesting that the functional benefit of f-URS in the short-term may be related to the avoidance of parenchymal trauma.

### 3.4. CKD Stage Distribution and Clinical Significance

The distribution of Chronic Kidney Disease (CKD) stages was evaluated to assess the clinical impact of the interventions (Table 5). At baseline, the majority of patients in both the mPCNL (92.3%) and f-URS (91.7%) groups were classified as CKD Stage 2. No patients in either group presented with CKD Stage 3 or higher at baseline.

Postoperatively, the relief of stone-related obstruction resulted in a biological improvement of renal filtration across both cohorts. At 1 month, a notable shift toward CKD Stage 1 was observed: the proportion of patients with an eGFR ≥ 90 mL/min/1.73 m^2^ increased to 30.8% in the mPCNL group and 41.7% in the f-URS group. Despite this favorable trend, the study’s short follow-up period precludes conclusions regarding long-term functional stability. These findings are considered hypothesis-generating, reinforcing the need for longitudinal studies to determine whether these early shifts in CKD distribution translate into a reduced risk of long-term renal failure in the solitary kidney population.

## 4. Discussion

For patients with solitary kidneys, managing renal stones requires a careful balance between achieving stone clearance and optimizing the risk–benefit profile to prioritize nephron preservation. Unlike patients with two functional kidneys, those with a solitary kidney lack physiological reserve, meaning that any procedural complication or decline in filtration rate can lead to serious clinical outcomes. Therefore, the choice of surgical modality is a significant clinical decision where safety and functional preservation are as critical as stone-free rates.

Current EAU guidelines recommend PCNL and f-URS as preferred treatment options for renal stones [8,16,17]; however, the optimal approach for solitary kidney patients—particularly for stones sized 1.0–2.0 cm—remains controversial despite numerous studies [18,19]. While both modalities have been reported as safe in this population [12,13], conclusions remain inconsistent, largely because traditional outcome metrics (stone-free rates, operative time) fail to capture the unique vulnerabilities of these patients [20]. With advancements in endoscopic technology, renal stone management has become increasingly diversified [21,22]. In our study, the 3-month stone-free rates were comparable between groups (mPCNL 96.2% vs. f-URS 91.7%, *p* = 0.157), confirming that for the 1.0–2.0 cm size range, f-URS achieves equivalent clearance to mPCNL without compromising efficacy. This equivalence is particularly relevant for solitary kidney patients, in whom residual fragments carry higher risks of obstruction and infection that could precipitate acute kidney injury. Mean operative time was slightly longer in the f-URS group (68.5 ± 15.2 vs. 58.3 ± 12.7 min, *p* < 0.05), but this modest difference did not translate into adverse functional outcomes.

Safety profiles, however, differed notably between the two approaches. The f-URS group demonstrated a significantly lower mean hemoglobin decline (2.2 ± 0.9 vs. 5.7 ± 2.4 g/dL, *p* < 0.001), and all Grade II complications requiring blood transfusion occurred exclusively in the mPCNL cohort. This inherent risk of hemorrhage during percutaneous access creation is a well-documented concern, with studies reporting significant bleeding events even in large cohorts of solitary kidney patients [23,24].This reflects the fundamental advantage of the retrograde approach, which utilizes the natural urinary tract and avoids the parenchymal trauma inherent to percutaneous access. Additionally, the f-URS group had a shorter hospital stay (4.1 ± 1.1 vs. 7.8 ± 1.6 days, *p* < 0.001), facilitating a faster return to normal activities. However, residual fragments carry amplified significance in solitary kidneys. Recent evidence [25] demonstrates that 20 to 30% of patients with residual fragments experience stone-related events requiring re-intervention within 2 to 3 years, including obstruction, infection, and stone growth. In solitary kidneys, acute ureteral obstruction from steinstrasse may precipitate acute kidney injury without contralateral compensation, and stone-associated infections may rapidly progress to urosepsis with catastrophic consequences. Critically, all Grade II complications (*n* = 2, requiring blood transfusion) in our study occurred exclusively in the mPCNL cohort, highlighting that while both approaches achieve similar stone clearance, f-URS eliminates the severe bleeding risk inherent to percutaneous access.

The superior safety profile and renal functional preservation observed in the f-URS group cannot be attributed solely to the retrograde approach itself, but rather to the specific technical protocols employed in this study that were instrumental in achieving these favorable outcomes. Intrarenal pressure (IRP) control was achieved through standardized gravity-fed irrigation (60–80 cm height) with temporary cessation when excessive pelvic distension was visualized, maintaining IRP below the established 30 cmH_2_O safety threshold that prevents pyelovenous backflow, fornix rupture, and tubular injury. Recent systematic reviews [26] emphasize the importance of efficient stone dust management to prevent obstruction, and our disciplined pressure management likely contributed to the superior early eGFR recovery trajectory observed in the f-URS group. Routine ureteral access sheath (UAS, 13 Fr) placement in all f-URS cases created a low-resistance egress pathway for irrigant, stone fragments, and debris, serving as a pressure-release valve during active lithotripsy. Beyond facilitating multiple scope passages, this strategy directly addresses stone dust evacuation challenges and prevents dangerous pressure buildup. Successful UAS placement in 100% of cases without ureteral perforation reflects careful patient selection and meticulous technique. Strategic postoperative drainage with routine double-J stent placement (2–4 weeks) ensured adequate drainage despite postoperative edema, facilitated the passage of small residual fragments, and prevented ureteral stricture formation—all critical when managing the sole functioning kidney where obstruction tolerance is zero. For mPCNL patients, nephrostomy drainage was maintained for 24–48 h to ensure adequate hemostasis and drainage. These integrated technical strategies represent the procedural foundation that enables f-URS to achieve comparable stone clearance while delivering superior functional preservation—demonstrating that technical discipline can overcome anatomical limitations in solitary kidney management.

Assessment by Clavien–Dindo classification revealed no statistical difference in overall complication rates between groups. However, the distribution of complications was markedly different: both Grade II complications (requiring blood transfusion) occurred in the mPCNL group, while the f-URS group had zero severe bleeding events. The mean hemoglobin decline was significantly lower with f-URS (2.2 ± 0.9 vs. 5.7 ± 2.4 g/dL, *p* < 0.001), reflecting the fundamental distinction between approaches: mPCNL necessitates parenchymal tract creation with inevitable vascular injury, whereas f-URS leverages the natural urinary tract without parenchymal disruption. In solitary kidneys, even moderate blood loss may compromise renal perfusion and oxygen delivery to an already stressed nephron mass, potentially exacerbating acute kidney injury. The f-URS group also demonstrated significantly shorter hospitalization (3.2 ± 1.1 vs. 5.8 ± 1.9 days, *p* < 0.001), reducing nosocomial infection exposure and enabling a faster return to normal activities.

Renal function was rigorously assessed using serum creatinine (Scr) and eGFR calculated by the CKD-EPI 2021 equation [27,28], a method recommended by current guidelines for its superior accuracy compared to older formulas, particularly in patients with preserved renal function [14,15]. Measurements at baseline, 72 h, and 1 month postoperatively captured both acute changes and early recovery patterns. The mean preoperative Scr levels were comparable between groups (mPCNL 112.9 ± 34.5 vs. f-URS 110.4 ± 31.3 μmol/L, *p* = 0.543). Postoperatively, both groups showed functional improvement due to the relief of stone-related obstruction. However, the f-URS group exhibited a more robust early recovery trajectory. Significant between-group differences emerged at 72 h (mPCNL 109.1 ± 29.4 vs. f-URS 104.6 ± 25.5 μmol/L, *p* = 0.041) and at 1 month (mPCNL 103.4 ± 28.3 vs. f-URS 93.7 ± 24.1 μmol/L, *p* = 0.008). The absolute change in Scr (ΔScr) from baseline was significantly greater in the f-URS group both at 72 h (f-URS 6.1 ± 4.64 vs. mPCNL 3.6 ± 2.45 μmol/L, *p* = 0.024) and at 1 month (f-URS 15.4 ± 7.96 vs. mPCNL 8.7 ± 4.23 μmol/L, *p* < 0.001). Correspondingly, the absolute improvement in eGFR (ΔeGFR) was significantly larger in the f-URS group at 72 h (7.6 ± 3.73 vs. 6.1 ± 5.32 mL/min/1.73 m^2^, *p* = 0.044) and at 1 month (16.7 ± 4.7 vs. 15.4 ± 5.2 mL/min/1.73 m^2^, *p* = 0.023). While the 1.3 mL/min/1.73 m^2^ difference at one month is modest, in a solitary kidney, even incremental preservation of function may contribute to overall renal resilience. These findings align with recent systematic reviews in solitary kidney populations [29,30], confirming superior functional preservation with ureteroscopy compared to PCNL.

The trajectory of eGFR change deserves particular attention. Singh et al. [31] demonstrated that eGFR slope analysis across CKD stages provides valuable prognostic information, with steeper negative slopes correlating with worse long-term outcomes. In our cohort, the f-URS group demonstrated consistently greater eGFR improvement from baseline to 1 month (mean improvement 16.7 ± 4.7 mL/min/1.73 m^2^), reflecting a more robust early functional recovery pattern compared to the mPCNL group. In contrast, the mPCNL group showed more modest improvement (15.4 ± 5.2 mL/min/1.73 m^2^, *p* = 0.023 from baseline). This difference in early functional trajectory—more robust improvement versus moderate improvement—may have significant implications for long-term renal preservation, particularly in patients with baseline CKD stage 3 or higher where functional reserve is already compromised. Consistent with our explicit disclaimer, these findings illustrate early recovery patterns and cannot be used to infer long-term renal functional stability or the long-term eGFR slope.

The mechanism underlying post-procedural eGFR improvement likely reflects the resolution of chronic partial obstruction caused by the index stone, with consequent improvement in glomerular hemodynamics and tubular function. However, the superior recovery magnitude in the f-URS cohort suggests that the avoidance of parenchymal trauma confers additional protective benefits beyond simple stone removal. Whether mPCNL adversely affects renal function in solitary kidneys remains debated. Streem et al. found no change in the serum creatinine in five patients one month after PCNL [32], while Akman et al. reported that 6.8% experienced worsening renal function [33]. Our retrospective data support that f-URS offers superior functional preservation for appropriately selected stone sizes.

This study exclusively employed holmium:YAG (Ho: AG) laser (20–40 W for mPCNL, 20–30 W for f-URS), which remains the global standard for urological lithotripsy. However, recent comparative analyses [34] demonstrate that newer laser technologies (thulium fiber laser [TFL], pulsed thulium:YAG) offer superior ablation efficiency, reduced operative time, better dust control, and minimal retropulsion compared to Ho:YAG. This represents an important limitation of our study. The exclusive use of Ho:YAG means that the findings may not fully generalize to contemporary practice employing newer platforms that could potentially further enhance the f-URS outcomes through faster stone ablation, reduced irrigation volumes (thus lower cumulative IRP exposure), and more complete fragment evacuation. Future comparative studies incorporating these newer technologies in solitary kidney populations are warranted to determine whether their theoretical advantages translate into measurably improved clinical outcomes such as reduced acute kidney injury rates, faster functional recovery, and lower re-intervention rates.

This study has several important limitations that should be considered when interpreting the results. First, the non-randomized, consecutive treatment allocation based on surgeon and patient preference, alongside anatomical considerations, introduces potential selection bias. Although our baseline analysis showed no statistically significant differences in stone size, density, or patient demographics between the two groups, and we employed ANCOVA to adjust for known baseline variables, unmeasured confounders inherent to retrospective observational designs may still influence the reported outcomes. Consequently, our findings should be interpreted as reflecting real-world clinical practice rather than a controlled experimental comparison. Second, the relatively small sample size (*n* = 26 f-URS, *n* = 24 mPCNL) limits statistical power to detect differences in rare but serious complications such as urosepsis or permanent renal loss. Third, the follow-up period (1 month for renal function and 3 months for stone-free rate), while adequate for assessing acute and early outcomes, is insufficient to capture delayed complications such as ureteral strictures, chronic functional deterioration, or stone recurrence. Longer-term follow-up (12–24 months) would strengthen conclusions regarding the durability of functional outcomes. Fourth, several technical and methodological factors limit our findings. Our exclusive use of the Holmium:YAG laser restricts generalizability to centers using newer technologies like thulium fiber laser (TFL), which may offer different safety and efficiency profiles. Additionally, we did not utilize real-time intrarenal pressure monitoring devices, which would provide more precise quantification of pressure exposure and its relationship to functional outcomes. Comprehensive metabolic stone evaluation and long-term recurrence prevention strategies were also not systematically addressed.

Despite these limitations, this study provides hypothesis-generating evidence that f-URS may offer a superior early safety and functional recovery profile for 1.0–2.0 cm solitary renal stones compared to mPCNL.

## 5. Conclusions

In this retrospective study of solitary kidney patients with 1.0–2.0 cm renal stones, both f-URS and mPCNL achieved comparable 3-month stone-free rates. Our preliminary data suggest that f-URS may offer a superior safety profile, characterized by reduced hemoglobin loss and shorter hospital stays. Furthermore, f-URS was associated with a more pronounced early renal functional recovery trajectory within the first month postoperatively. These findings suggest that f-URS represents a viable nephron-sparing option that may be considered for this high-risk population. However, given the study’s observational nature and limited sample size, these results should be interpreted as hypothesis-generating rather than definitive clinical recommendations. Prospective, randomized controlled trials with extended follow-up are essential to confirm these potential functional advantages and assess their long-term durability.

## Figures and Tables

**Table 1:** Demographic and clinical data of the included population.

Parameter	mPCNL *n* = 26	f-URS *n* = 24	*p*
Demographic characteristics			
Age (years), mean (SD)	45.3 ± 9.8	40.8 ± 7.2	0.356
Gender (male/female)	9/17	14/10	0.782
BMI (kg/m^2^), mean (SD)	23.2 ± 5.2	22.5 ± 4.8	0.620
Creatinine (μmol/L), mean (SD)	112.9 ± 34.5	110.4 ± 31.3	0.543
Comorbidities, *n* (%)			
Hypertension	2 (7.6%)	2 (8.3%)	0.579
Diabetes mellitus	2 (7.6%)	1 (4.2%)	0.246
Coronary heart disease	1 (3.8%)	1 (4.2%)	0.438
Stone characteristics			
Stone density (HU), mean (SD)	765.8 ± 160.2	785.5 ± 174.1	0.585
Stone diameter (mm), mean (SD)	20.2 ± 1.4	19.8 ± 1.8	0.356
Site of stones (left), *n* (%)	13 (50.0%)	11 (45.8%)	0.858
Preoperative infection (Yes), *n* (%)	5 (19.2%)	3 (12.5)	0.344
Kidney characteristics			
Functional solitary kidney	7 (26.9%)	5 (20.8%)	0.362

mPCNL—mini-Percutaneous nephrolithotripsy; f-URS—flexible ureteroscopy; BMI—mean body mass index; HU—Hounsfield units.

**Table 2:** Perioperative treatment results.

Parameter	mPCNL *n* = 26	f-URS *n* = 24	*p*
Operation time (min)	67.4 ± 16.9	78.4 ± 18.3	0.106
Mean decline in Hb level (g/dL)	5.7 ± 2.4	2.2 ± 0.9	<0.001
Length of hospitalization (days)	7.8 ± 1.6	4.1 ± 1.1	<0.001
Stone-free rate at 3 month (%)	25 (96.2)	22 (91.7)	0.157
Complication rate (%)	6 (23.1)	5 (20.8)	0.656
Grade I	4	5	
Grade II	2	0	
Grade >III	0	0	

mPCNL—mini-Percutaneous nephrolithotripsy; f-URS—flexible ureteroscopy.

**Table 3:** Perioperative renal function changes.

Parameter	mPCNL *n* = 26	f-URS *n* = 24	*p*
**Baseline**			
Serum creatinine (μmol/L)	112.9 ± 34.5	110.4 ± 31.3	0.543
eGFR (mL/min/1.73 m^2^)	75.9 ± 9.7	72.3 ± 11.8	0.476
**Postoperative values**			
Scr at 72 h (μmol/L)	109.1 ± 29.4	104.6 ± 25.5	0.041
eGFR at 72 h (mL/min/1.73 m^2^)	81.0 ± 11.6	80.8 ± 12.5	0.033
Scr at 1 month (μmol/L)	103.4 ± 28.3	93.7 ± 24.1	0.008
eGFR at 1 month (mL/min/1.73 m^2^)	87.3 ± 6.7	88.2 ± 7.4	0.022
**Change from baseline (Δ)**			
ΔScr at 72 h (μmol/L)	−3.6 ± 2.45	−6.1 ± 4.64	0.024
ΔeGFR at 72 h (mL/min/1.73 m^2^)	+ 6.1 ± 5.32	+7.6 ± 3.73	0.044
ΔScr at 1 month (μmol/L)	−8.7 ± 4.23	−15.4 ± 7.96	<0.001
ΔeGFR at 1 month (mL/min/1.73 m^2^)	+15.4 ± 5.2	+16.7 ± 4.7	0.023

mPCNL—mini-Percutaneous nephrolithotripsy; f-URS—flexible ureteroscopy; Scr—serum creatinine; eGFR—the estimated glomerular filtration rate.

**Table 4:** Adjusted between-group differences in renal function changes (f-URS vs. mPCNL).

Outcome Measure	Unadjusted Mean Difference	Adjusted Mean Difference	95% Confidence Interval (CI)	*p*-Value
ΔScr at 72 h (μmol/L)	−2.50	−2.52	[−4.66, −0.34]	0.024
ΔeGFR at 72 h (mL/min/1.73 m^2^)	1.50	1.45	[0.04, 2.96]	0.044
ΔScr at 1 month (μmol/L)	−6.70	−6.75	[−10.31, −3.09]	<0.001
ΔeGFR at 1 month (mL/min/1.73 m^2^)	1.30	1.28	[0.19, 2.41]	0.023

Scr—serum creatinine; eGFR—the estimated glomerular filtration rate. Adjusted for baseline stone diameter, stone density, and baseline renal function (eGFR/Scr).

**Table 5:** Distribution of Chronic Kidney Disease (CKD) stages at baseline and 1-month follow-up.

CKD Stage (eGFR Range)	mPCNL (*n* = 26) Baseline	f-URS (*n* = 24) Baseline	mPCNL (*n* = 26) 1 Month	f-URS (*n* = 24) 1 Month
Stage 1 (≥90 mL/min/1.73 m^2^)	2 (7.7%)	2 (8.3%)	8 (30.8%)	10 (41.7%)
Stage 2 (60–89 mL/min/1.73 m^2^)	24 (92.3%)	22 (91.7%)	18 (69.2%)	14 (58.3%)
Stage 3 (30–59 mL/min/1.73 m^2^)	0 (0%)	0 (0%)	0 (0%)	0 (0%)

mPCNL—mini-Percutaneous nephrolithotripsy; f-URS—flexible ureteroscopy.

## Data Availability

The datasets used in this study can be obtained from the corresponding author on reasonable request.

## Supplementary Materials

The following supporting information can be downloaded at: https://www.mdpi.com/article/10.3390/jcm15052089/s1, Table S1: Selection of surgical method.

## Abbreviations

The following abbreviations are used in this manuscript:

mPCNL	Mini-percutaneous nephrolithotomy
f-URS	Flexible ureteroscopy
BMI	Body mass index
HU	Hounsfield unit
Scr	Serum creatinine
eGFR	The estimated glomerular filtration rate
CKD	Distribution of Chronic Kidney Disease
