# Supplementary material for: Comparison of Mini-Percutaneous Nephrolithotomy and Flexible Ureteroscopy for Treating 1–2 cm Single Stones in Solitary Kidney: Outcomes and Renal Function Impact
Source: J Clin Med. 2026 Mar 9;15(5):2089. doi: 10.3390/jcm15052089 (PMC12986005; doi:10.3390/jcm15052089)
Supplement: Supplementary file 1 [file jcm-15-02089-s001.zip › jcm-4171434-supplementary.pdf]

Supplementary Table S1 Selection of surgical method

Patient Number	Surgeon Preference	Patient Preference	Anatomical Considerations	Stone Characteristics	Institutional Availability	Shared decision-making
1	mPCNL	mPCNL	Functional solitary kidney	a single renal stone	YES	mPCNL
2	f-URS	f-URS	nephrectomy	a single renal stone	YES	f-URS
3	f-URS	f-URS	Functional solitary kidney	a single renal stone	YES	f-URS
4	mPCNL	mPCNL	nephrectomy	a single renal stone	YES	mPCNL
5	f-URS	f-URS	nephrectomy	a single renal stone	YES	f-URS
6	mPCNL	mPCNL	nephrectomy	a single renal stone	YES	mPCNL
7	f-URS	f-URS	nephrectomy	a single renal stone	YES	f-URS
8	f-URS	f-URS	nephrectomy	a single renal stone	YES	f-URS
9	mPCNL	mPCNL	Functional solitary kidney	a single renal stone	YES	mPCNL
10	f-URS	f-URS	nephrectomy	a single renal stone	YES	f-URS
11	mPCNL	mPCNL	nephrectomy	a single renal stone	YES	mPCNL
12	f-URS	f-URS	Functional solitary kidney	a single renal stone	YES	f-URS
13	mPCNL	mPCNL	Functional solitary kidney	a single renal stone	YES	mPCNL
14	mPCNL	mPCNL	nephrectomy	a single renal stone	YES	mPCNL
15	f-URS	f-URS	congenital absence of one kidney	a single renal stone	YES	f-URS
16	mPCNL	mPCNL	nephrectomy	a single renal stone	YES	mPCNL
17	f-URS	f-URS	nephrectomy	a single renal stone	YES	f-URS
18	f-URS	f-URS	nephrectomy	a single renal stone	YES	f-URS
19	mPCNL	mPCNL	Functional solitary kidney	a single renal stone	YES	mPCNL
20	f-URS	f-URS	nephrectomy	a single renal stone	YES	f-URS
21	mPCNL	mPCNL	congenital absence of one kidney	a single renal stone	YES	mPCNL
22	mPCNL	mPCNL	nephrectomy	a single renal stone	YES	mPCNL
23	mPCNL	mPCNL	nephrectomy	a single renal stone	YES	mPCNL
24	f-URS	f-URS	Functional solitary kidney	a single renal stone	YES	f-URS
25	f-URS	f-URS	nephrectomy	a single renal stone	YES	f-URS
26	mPCNL	mPCNL	Functional solitary kidney	a single renal stone	YES	mPCNL
27	f-URS	f-URS	nephrectomy	a single renal stone	YES	f-URS
28	f-URS	f-URS	congenital absence of one kidney	a single renal stone	YES	f-URS
29	mPCNL	mPCNL	nephrectomy	a single renal stone	YES	mPCNL

30	f-URS	f-URS	nephrectomy	a single renal stone	YES	f-URS
31	f-URS	f-URS	nephrectomy	a single renal stone	YES	f-URS
32	mPCNL	mPCNL	nephrectomy	a single renal stone	YES	mPCNL
33	f-URS	f-URS	Functional solitary kidney	a single renal stone	YES	f-URS
34	f-URS	f-URS	nephrectomy	a single renal stone	YES	f-URS
35	mPCNL	mPCNL	congenital absence of one kidney	a single renal stone	YES	mPCNL
36	f-URS	f-URS	nephrectomy	a single renal stone	YES	f-URS
37	mPCNL	mPCNL	Functional solitary kidney	a single renal stone	YES	mPCNL
38	mPCNL	mPCNL	nephrectomy	a single renal stone	YES	mPCNL
39	f-URS	f-URS	congenital absence of one kidney	a single renal stone	YES	f-URS
40	mPCNL	mPCNL	nephrectomy	a single renal stone	YES	mPCNL
41	f-URS	f-URS	nephrectomy	a single renal stone	YES	f-URS
42	mPCNL	mPCNL	nephrectomy	a single renal stone	YES	mPCNL
43	mPCNL	mPCNL	nephrectomy	a single renal stone	YES	mPCNL
44	mPCNL	mPCNL	Functional solitary kidney	a single renal stone	YES	mPCNL
45	f-URS	f-URS	Functional solitary kidney	a single renal stone	YES	f-URS
46	mPCNL	mPCNL	nephrectomy	a single renal stone	YES	mPCNL
47	mPCNL	mPCNL	nephrectomy	a single renal stone	YES	mPCNL
48	mPCNL	mPCNL	nephrectomy	a single renal stone	YES	mPCNL
49	f-URS	f-URS	nephrectomy	a single renal stone	YES	f-URS
50	mPCNL	mPCNL	nephrectomy	a single renal stone	YES	mPCNL

mPCNL mean *mini-Percutaneous nephrolithotripsy*, *f-URS* mean *flexible ureteroscopy*
